# Anthropometry, Body Composition, and Performance in Sport-Specific Field Test in Female Wheelchair Basketball Players

**DOI:** 10.3389/fphys.2018.00568

**Published:** 2018-05-30

**Authors:** Valentina Cavedon, Carlo Zancanaro, Chiara Milanese

**Affiliations:** Laboratory of Anthropometry and Body Composition, Department of Neurosciences, Biomedicine and Movement Sciences, University of Verona, Verona, Italy

**Keywords:** classification, disabled, mixed teams, physique, fat mass

## Abstract

Data on the physical and performance characteristics of female wheelchair basketball (WB) players are scarce. In several countries female WB players train and compete with male players on mixed teams due to the limited total population of players, which would otherwise lead to large territorial spread for each team. Any differences in terms of physical characteristics and/or WB skill proficiency between male and female WB players would be relevant to team performance in mixed teams. This work examined anthropometry, body composition, and performance in a set of sport-specific field tests in a sample of 13 female WB players representing about 40% of the eligible population in Italy across a range of functional point scores (Point). Point is assigned on an ordinal scale from 1.0 (i.e., players with minimal functional potential) through to 4.5 (players with maximum functional potential). Our female sample was then compared against twice as many (*n* = 26) Point-matched (±0.5 points) male players. The two groups were similar for age (*P* = 0.191; effect size [*d*] = 0.2), self-reported duration of injury (*P* = 0.144, *d* = 0.6), WB experience (*P* = 0.178, *d* = 0.5), and volume of training (*P* = 0.293, *d* = 0.4). The large majority of measured linear anthropometric variables (10/13) were lower in female players than males (0.001 < *P* ≤ 0.041). Skinfold-estimated percent body fat was higher (+7.6%) in females (30.7 ± 6.0%; *P* < 0.001, *d* = 1.3). Mean performance was worse in female than in males in six out of seven sport-specific field tests, scores being significantly lower in females for the maximal pass (7.5 ± 2.0 m for females vs. 10.4 ± 2.8 m for males; *P* = 0.002, *d* = 1.2) and suicide tests (55.8 ± 6.4 s for females vs. 45.4 ± 6.7 s for males; *P* < 0.001, *d* = 1.6). When performance in subgroups of females (*n* = 9) chosen across a range of Point was compared with that of males assigned 1.0 or 1.5 Point less (each *n* = 9), performance differences between male and female WB players were partially and completely eliminated, respectively. This work contributed new data for characterizing the physique and performance of female WB players. Further, the results suggested that when male and female athletes compete together in mixed teams, a 1.5 points subtraction from female players is needed to match the real gender difference in performance.

## Introduction

Wheelchair basketball (WB) represents one of the most popular and inclusive adapted team sports for athletes with physical impairments being practiced by about 100,000 players worldwide. WB competitions are open to male and female players at international, national, recreational, collegiate, and junior levels in nearly a 100 countries around the world. WB applies most major rules and scoring from the sport of running basketball, but introduces some special adjustments considering the presence of subjects with different impairments and the use of the wheelchair in the game.

Wheelchair basketball is reserved for athletes with a range of permanent lower limb impairments that prevent running, jumping, and pivoting at speed and with the control, safety, stability, and endurance of an able-bodied player. WB is therefore reserved for athletes with different types of impairments (e.g., spinal cord injury, amputation, and poliomyelitis) and severity of impairment (e.g., spinal cord level of the lesion or complete/incomplete spinal cord injury). Given the wide range of activity-limiting impairments included, players are classified according to the extent to which their impairment impacts on their WB performance. Based on such a classification system, players are assigned a functional point score (hereinafter Point) from Point 1.0 (i.e., players with minimal functional potential) through to Point 4.5 (players with maximal functional potential) on an ordinal scale with 0.5-point increments (International Wheelchair Basketball Federation [IWBF], 2014). In other words, players with higher functional limitation (e.g., a player with complete spinal cord injury at the thoracic level) are assigned to the Point 1.0 category, while players with smaller functional limitation (e.g., a player with a unilateral lower limb amputation) are assigned to the Point 4.5 category. To promote inclusion, in the Italian Young Wheelchair Basketball Championship players may be assigned to a Point 0.5 category to include individuals meeting both the general IWBF eligibility criteria and an additional criterion of permanent physical impairment resulting in the substantial loss of function in one or both upper extremities (e.g., tetraplegia) (Italian Wheelchair Basketball Federation, 2017). In order to ensure that all eligible players have an opportunity to be an integral member of the team and in order to make competition between teams balanced, each team must play to a specific team point maximum. That is, during competition each team is allowed to put into play five players with a maximum totaling 14 points at any given time.

Opportunities for females to participate in WB have been increasing at both the international and national levels since the seventies. Indeed, women’s WB was first played at the 1968 Paralympic Games in Tel Aviv, while men’s WB has been a part of the Summer Paralympic Games since 1960 in Rome. Since that time, WB has been enjoying great success within the Paralympic movement and the number of elite male and female athletes performing in Paralympic events has increased. In several national championships, female WB players train and compete with male players in mixed teams due to the limited total population of players, which would otherwise lead to large territorial spread for each team. This raises the question as to whether differences in terms of physical characteristics and/or WB skill proficiency between male and female WB players are relevant to team performance. While differences in physique and performance between male and female able-bodied basketball players may be deemed as obvious, it should be kept in mind that WB athletes represent a special population where physical and performance characteristics are connected to residual functional capacity in a complex individual way making evaluation of sex-related differences cumbersome. When male and female athletes compete together on the same team a modified system of classification is adopted. When female athletes play on a mixed gender team, 1.0 or 1.5 points are currently subtracted from each female WB player’s point value, leaving extra points for use in selecting the other team members with higher point values. It is understood that this point subtraction is given to compensate for the possible difference in WB performance between male and female players and to encourage more female players into the sport. However, such provisions are empirical and not made according to an “evidence-based classification system through research” as recommended by the International Paralympic Committee [IPC] (2007). Therefore, a key question is whether point subtraction is actually needed (and to what extent) in order to match the real difference in performance between male and female WB players. Detailed information on the physical characteristics of female WB players and their performance in standardized field tests are lacking, as is any comparison between female and male WB players with regards to physical characteristics and performance. As a matter of fact, most of the available literature on WB focused on male players only (Vanlandewijck et al., 1994; Malone et al., 2002; Bernardi et al., 2010; De Lira et al., 2010; Molik et al., 2010; De Witte et al., 2015) or male and female players considered as a whole (De Groot et al., 2012; Cavedon et al., 2015; Granados et al., 2015; Weissland et al., 2015). A very limited number of studies investigated WB female players as an individual group (Curtis and Black, 1999; Vanlandewijck et al., 2004; Molik et al., 2009; Gómez et al., 2014, 2015). The issue of sex-related differences in WB performance profiles has also been scarcely explored (Vanlandewijck et al., 2004; Molik et al., 2009; Gómez et al., 2014). A further limitation of previous work is that only game-related statistics in elite players were investigated with no reference to performance in standardized field tests.

In the literature there are a few studies reporting data on the anthropometric and body composition characteristics of athletes with disabilities in WB (Cavedon et al., 2015; Granados et al., 2015) as well as some of the other Paralympic sports, e.g., swimming (Dingley et al., 2015), throwing (Spathis et al., 2015), athletics (Connick et al., 2016), ice sledge hockey (Molik et al., 2012), and rowing (Porto et al., 2008). In WB the papers by Cavedon et al. (2015) and Granados et al. (2015), highlighted that the sitting height, along with other upper body linear anthropometric variables, are especially relevant to WB performance, reporting that higher values in the sitting height could give some advantage in WB performance (e.g., throwing or passing the basketball ball). Further, in Paralympic swimming, Dingley et al. (2015) highlighted some traits of the physical profile (e.g., large chest girth, short arm span, high stature, and low skinfolds) of Paralympic swimmers, which would be advantageous to swimming performance. Intriguingly, the profile they reported to be beneficial to the swimming performance varied according to the gender of athletes and the severity of their impairment. In WB and in the other Paralympic sports, the analysis of the anthropometric and body composition profiles of athletes with physical disabilities could help in the selection of key anthropometric measures to be used by coaches in the design and implementation of training programs in order to improve the probability of success. What is more, a detailed delineation of the physical profile of WB players according to gender is expected to provide a greater understanding in the performance profiles of male and female players.

There is therefore a need to enhance the current literature with scientific data on the physique and the performance characteristics of female WB players and to evaluate whether (and to what extent) sex impacts on skill proficiency of male and female players. Taking into account the above issues, this study had a threefold aim: first, to examine a sample of female WB players in order to characterize their anthropometry, body composition, and performance in sport-specific field tests; second, to explore sex-related differences in the above variables by comparing female WB players with twice as many male Point-matched WB players; third, to assess the effectiveness of subtracting points to compensate for sex-related differences in performance using sport-specific field tests as the reference. The data from the present study would be useful as a reference for female WB players and complementary to the existing literature on WB. They would also promote an appropriate evidence-based classification system for athletes taking into account both sex and functional ability.

## Materials and Methods

### Participants

Sample size was estimated “*a priori*” using G^∗^Power (Faul et al., 2009) based on data of performance in one primary outcome (i.e., the maximal pass test) of a sample of female players (*n* = 7) (Cavedon et al., 2015). Setting the type I error at α = 0.05, the effect size at *d* = 1.0, and allocation ratio N2/N1 equal to 0.5 the minimum sample size required for having a 80% power (i.e., β = 0.20) was 13 and 26 subjects for the female and male group, respectively. The inclusion criteria were age > 16 years and at least 1 year of WB experience at a competitive level. In the 2013/2014 competitive season, 50 teams competed in the various Italian Wheelchair Basketball Championships (A1 League, A2 League, B League and Young); of these, 22 had at least one female player on the team. Thirteen female WB players playing in eight different teams, representing about 40% of the eligible population, volunteered to participate in this study. Age, sex, and Point were obtained for all players from the database on the Italian Wheelchair Basketball Federation (2014) website. The participant group (**Table 1**) encompassed players from almost every Point with 1–3 participants in each class. The group typified the range of physical impairment found in the general female WB population by type and degree of severity. The disabilities of the female WB players included spinal cord injury (complete/incomplete paraplegia, *n* = 6), spina bifida (*n* = 2), phocomelia (*n* = 1), lower extremity poliomyelitis (*n* = 2), spastic tetraparesis (*n* = 1), and unilateral above-knee amputation (*n* = 1).

**Table 1:** Characteristics of wheelchair basketball (WB) players.

Female participant	Point	WB exp (y)	League	Male participant	Point	WB exp (y)	League
1	1.0	7	B	1	1.0	4	B + Y
				2	1.0	5	B + Y
2	2.5	6	A1	3	3.0	1	B
				4	2.5	9	B + Y
3	3.0	8	B	5	3.5	23	B
				6	2.5	4	Y
4	2.5	6	A1+Y	7	2.5	7	B + Y
				8	1.5	4	B + Y
5	4.0	1	B	9	3.5	3	B + Y
				10	4.0	7	B + Y
6	2.0	1	B	11	2.0	7	Y
				12	2.0	8	Y
7	2.0	7	B	13	2.0	4	B
				14	1.5	11	Y
8	3.0	2	Y	15	3.0	14	B + Y
				16	2.5	7	B + Y
9	2.0	4	B + Y	17	2.0	9	B + Y
				18	2.5	8	B
10	1.5	9	B + Y	19	1.5	2	Y
				20	1.5	4	B + Y
11	3.0	8	B + Y	21	3.0	8	B + Y
				22	3.5	15	B
12	0.5	9	Y	23	0.5	7	Y
				24	0.5	10	Y
13	0.5	5	Y	25	0.5	10	Y
				26	0.5	5	Y

*Female participants (age range: 16–43 years) were each matched to two male participants (age range: 16–59 years) according to participant number (i.e., female participant #1 matched with male participants #1 and #2, etc.). A1, Italian A1 League Wheelchair Basketball Championship; A2, Italian A2 League Wheelchair Basketball Championship; B, Italian B League Wheelchair Basketball Championship; Y, Italian Young Wheelchair Basketball Championship; Point, functional point score; WB exp, wheelchair basketball experience.*

In order to assess the effects of sex on physical and performance variables, each female WB player in the participant group was matched with two male WB players recruited from four different WB teams on the basis of their assigned Point (±0.5). Point is a key parameter utilized by coaches to select players for a competitive match. The median Point value (interquartile range) was 2.0 (1.75) and 2.0 (1.72) in the female and male groups, respectively (*P* = 0.904). The disabilities of male WB players comprised spinal cord injury (complete/incomplete paraplegia, *n* = 5), spina bifida (*n* = 7), lower extremity poliomyelitis (*n* = 4), spastic tetraparesis (*n* = 4), spastic paraparesis (*n* = 1), spastic diplegia (*n* = 1), and cerebral palsy (*n* = 4). The main characteristics of the whole sample of female (*n* = 13) and male (*n* = 26) WB players participating in this study are summarized in **Table 1**.

In order to assess what functional point reduction (i.e., 1.0 or 1.5) is more suitable to compensate for sex-related differences in performance two further analyses were conducted in subgroups of players. First, performance was compared in female WB players (*n* = 9) assigned Point ≥ 1.5 and the same number of male WB players assigned 1.0 functional point less. The characteristics of this subgroup (Subgroup A) are reported in **Table 2**. Second, performance was compared in female WB player (*n* = 9) with Point ≥ 2.0 and the same number of male WB player assigned 1.5 points less. The characteristics of this subgroup (Subgroup B) are reported in **Table 3**. When matching male to female players, the male player best matching the corresponding female for age, duration of injury (DOI), and WB experience was preferred.

**Table 2:** Characteristics of WB players in Subgroup A.

Sex (M/F)	Point	DOI (y)	WB exp (y)	Sex (M/F)	Point	DOI (y)	WB exp (y)
F	1.5	20	9	M	0.5	20	7
F	2.0	6	1	M	1.0	4	4
F	2.0	7	4	M	1.0	17	5
F	2.5	9	6	M	1.5	21	4
F	2.5	16	6	M	1.5	16	4
F	3.0	17	8	M	2.0	20	7
F	3.0	22	2	M	2.0	18	8
F	3.0	14	8	M	2.0	18	9
F	4.0	2	1	M	3.0	2	1

*Female participants (age range: 16–43 years) assigned a range of Point (1.5–4.5) were each matched to a male WB player (age range: 16–26 years) assigned 1.0 Point less. F, female; M, male; Point, functional point score; y, years; DOI, duration of injury; WB exp, wheelchair basketball experience.*

**Table 3:** Characteristics of WB players in Subgroup B.

Sex (M/F)	Point	DOI (y)	WB exp (y)	Sex (M/F)	Point	DOI (y)	WB exp (y)
F	2.5	9	6	M	1.0	4	4
F	3.0	17	8	M	1.5	22	11
F	2.5	16	6	M	1.0	16	5
F	4.0	2	1	M	2.5	16	4
F	2.0	6	1	M	0.5	16	5
F	2.0	20	7	M	0.5	20	7
F	3.0	22	2	M	1.5	21	4
F	2.0	7	4	M	0.5	17	10
F	3.0	14	8	M	1.5	16	4

*Female participants (age range: 16–43 years) assigned a range of Points (2.5–4.0) were each matched to a male WB player (age range: 16–39 years) assigned 1.5 points less. F, female; M, male; Point, functional point score; y, years; DOI, duration of injury; WB exp, wheelchair basketball experience.*

The protocol conformed to the Declaration of Helsinki (revised in 2008). The Institutional Review Board at the University of Verona approved the study protocol. The study had full ethical approval and all participants gave their written informed consent.

### Anthropometry and Body Composition Assessment

Body circumferences were measured with a fiberglass Gulick Anthropometric Tape (Mabis Healthcare, Waukegan, IL, United States) at the upper arm (relaxed), the forearm, the wrist and the waist site. The following body dimensions were measured with a Harpenden anthropometer (Holtain, Ltd., Crymych, Pembrokeshire, United Kingdom) according to conventional criteria and measuring procedures (Lohman et al., 1988): shoulder-elbow length, elbow-wrist length, thigh length, transverse chest width, anterior–posterior chest depth, elbow width, and wrist width. Stature is difficult to measure with accuracy in athletes with disability because of the underlying pathology. In this study, The authors adopted an ecological approach by measuring the height of the player on his/her own basketball wheelchair assuming this to be a proxy of the player’s actual height during play. Following to a previous study (Cavedon et al., 2015), two measurements were taken: (1) the sitting height (SitH1), measured as the vertical distance from the vertex of the head to the floor and (2) the maximal vertical reach from a seated position (SitH2) measured as the maximal distance from the tip of the dactylion III to the floor, with the upper arms extended overhead as high as possible.

Skinfold thickness was measured to the nearest 0.1 mm with a Harpenden caliper (Gima, Milan, Italy) at the triceps, biceps, subscapular, and suprailiac sites according to standard procedures (Lohman et al., 1988). Duplicate readings were taken at each site, and the average of the two was recorded. If the two readings differed by more than 2 mm a third one was taken, and the closest two were averaged. The sum of the four skinfold measurements was used as an estimate of body density according to the sex- and age- specific Durnin-Womersley equation (Durnin and Womersley, 1974) as previously reported in Paralympic sitting athletes (Bernardi et al., 2012). Body density was then transformed into body fat percentage (%FM) according to Siri (1961).

All the participants completed the measures established in the study protocol. All anthropometric and body composition measurements were taken by the same expert operator (VC) who has been taking anthropometric measurements in subjects with physical disabilities since 5 years. For reliability, the technical error of measurement (TEM) was computed with the following formula (Ulijaszek and Lourie, 1994):

$$\text{TEM}\;\text{=}\;\sqrt{(\sum {\text{d}}^{\text{2}})\text{/2N}}$$

where Σd^2^ is the summation of deviations raised to the second power and N is the number of subjects measured. The absolute TEM was transformed into relative TEM (rTEM) in order to obtain the error expressed as a percentage corresponding to the total average of the variable to be analyzed, with the following formula:

$$\text{rTEM}\;\text{=}\;\frac{\text{TEM}\times \text{100}}{\text{VAT}}$$

where VAT represents the variable average value, i.e., the arithmetic mean of the mean between both measurements obtained from each subject for the same anthropometrical measure. The TEMs were below 1% for all lengths/breadths/girths and below 3.5% for all skinfolds recorded in this study and therefore within the acceptable limits (i.e., <5.0% for skinfold thickness and <1.0% for all other measures) (Gore et al., 2000).

### Assessment of Performance

**FIGURE 1:**
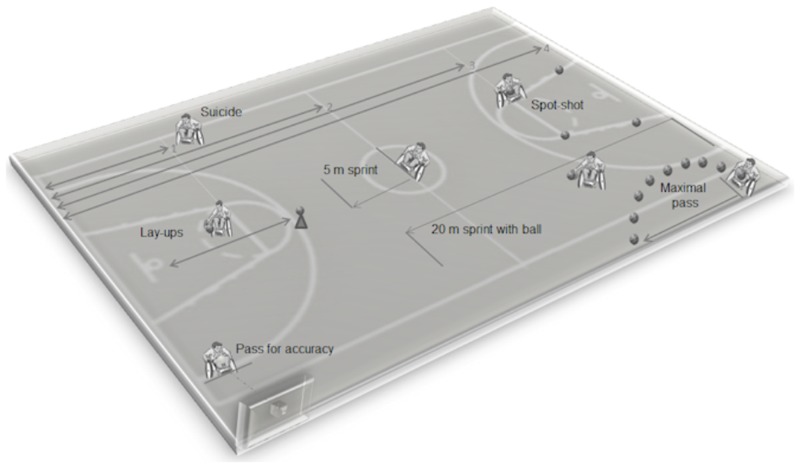
Layout of sport-specific field tests adopted from Cavedon et al. (2015).

Performance assessment included a range of sport-specific field tests exploring speed, ball handling, shooting, passing, and endurance (**Figure 1**). All tests were performed according to De Groot et al. (2012) with slight modifications. Field tests were performed following the teams’ usual warm-up on a basketball court in the gym either during a national meeting or during one of the usual on-court training session. Each participant used his own personal wheelchair. For the 5 m sprint test, the player started from a stationary position and pushed for a distance of 5 m as quickly as possible. For the 20 m with ball test, the player started with a ball from a stationary position and pushed for a distance of 20 m as fast as possible, following the FIPIC rules for bouncing. The score was the time taken to complete the 20 m distance. For the suicide test, the player positioned himself with the front wheels behind the baseline, pushing first to the foul line and back, then to the half line and back, then to the far foul line and back, then to the far baseline and back. The total time to complete these distances was the score. In the speed-related tests (5 m sprint, 20 m sprint, and suicide), the player started sprinting on a starting sound (i.e., “three, two, one, go”). Time was manually recorded with a Digital Traceable Stopwatch (Traceable Products, Webster, TX, United States) starting when the front wheels crossed the start line and stopping when the front wheels crossed the finish line. For the maximal pass test, the player was positioned stationary with the front wheels behind the baseline, attempting to throw the basketball ball as far as possible. The distance between the baseline and where the ball first hits the floor was measured with a Fiberglass Blade Long Tape rule (Stanley Black & Decker Corporation, Inc., New Britain, CT, United States). In the pass for accuracy test the player was positioned behind a 4 m distance line and had to pass the basketball 10 times toward a 30 cm square target (with a 2 cm border) marked on the wall of the sports hall with a scotch paint masking tape. The center of the square was at 1.2 m above the ground. Any form of pass was acceptable. Players scored 3, 1, or 0 points depending on where the ball hit the target. The score was the sum of the points from the 10 passes (range: 0–30). For the lay-ups tests, the players started with the basketball behind the 3-point line aiming to score as many lay-ups as possible within a minute. After each lay-up participants were asked to go back to the 3-point line and pick-up the ball from a cone. Depending on where the ball hit the scoring board, players scored 3 points (when the shot was a hit), 1 point (when the ball touched the ring but was not a hit) or 0 points (when the ball did not touch the ring at all). For the spot-shot test, the player had to perform five shots from four different positions around the lane, two at the top of the lane. Players scored 3 points (when the shot was a hit), 1 point (when the ball touched the ring but was not a hit) or 0 points (when the ball did not touch the ring at all). The score was the sum of the points of the 20 shots.

### Statistical Analysis

A cross-sectional comparative study design was adopted. Data were assessed for normality with the Kolmogorov–Smirnov test and transformed using the method described by Box and Cox (1964) where necessary. The Box and Cox transformation provides an algorithm through which the optimal value of the transformation parameter λ is selected by the method of maximum likelihood for reducing heterogeneity of error that permits the assumption of equal variance to be met. The Levene’s test was performed to assess the equality of variance of data. Descriptive statistics (mean and standard deviation) were computed for all variables using standard procedures. The *t*-test for independent samples (two-tailed) was used to assess sex differences in WB performance, anthropometry, and body composition between the whole sample of female WB players (*n* = 13) and their Point-matched male counterparts (*n* = 26). The Cohen’s *d* was used as a measure of the effect size (ES) and interpreted according to Cohen’s guidelines (Cohen, 1988) as small (*d* = 0.2), medium (*d* = 0.5), and large (*d* = 0.8). For categorical variables (Point), data were expressed as median (interquartile range) and comparisons performed with the Mann–Whitney test. The *t*-test for independent samples (two-tailed) was also used to assess sex differences in WB performance between the sub-group of female WB players and the sub-group of male WB players in both of the further two analyses on the effects of point reduction. Statistical power was evaluated using G^∗^Power Software 3.1 (Faul et al., 2009) on the basis of the observed effect sizes. All analysis was performed with SPSS v. 16.0 (IBM, Corp., Armonk, NY, United States). The alpha value was set at *P* ≤ 0.05.

## Results

The demographic, anthropometric, and sport-specific performance data of female WB players are summarized in **Table 4**. The differences in mean anthropometric and body composition characteristics of the female and male group of WB players are presented in **Table 5**. Female WB players and their Point-matched male counterparts had similar age (females: 25.3 ± 9.1 years, males 23.3 ± 11.1 years; *P* = 0.191, *d* = 0.2), self-reported duration of injury (females: 13.9 ± 6.4 years, males 19.3 ± 12.2 years; *P* = 0.144, *d* = 0.6), WB experience (females: 5.6 ± 2.8 years, males 7.5 ± 4.6 years; *P* = 0.178, *d* = 0.5), and volume of training (females: 5.2 ± 1.0 h/w, males: 4.8 ± 1.0 h/w; *P* = 0.293, *d* = 0.4). The upper arm (relaxed) and the waist circumferences, as well as thigh length, SitH1, SitH2, anterior–posterior chest width and upper body skinfold thickness were all similar in female and male WB players. Females showed significantly lower mean values for several upper body linear anthropometric variables namely, the forearm, wrist and waist circumference (-9.6%, *P* = 0.011, *d* = 0.9; -8.5%, *P* = 0.001, *d* = 1.2; and -9.6%, *P* = 0.041, *d* = 0.7, respectively), the shoulder-elbow and elbow-wrist length (-9.3%, *P* = 0.002, *d* = 1.1 and -8.8%, *P* < 0.001, *d* = 1.3, respectively) as well as the transverse chest (-11.4%, *P* = 0.003, *d* = 1.1), and the elbow and wrist width (-9.9%, *P* < 0.001, *d* = 1.2 and -9.4%, *P* = 0.002, *d* = 1.1, respectively). Female WB players had a significantly higher (+7.6%, *P* < 0.001, *d* = 1.3) percentage of body fat vs. males.

**Table 4:** Anthropometry, body composition, and performance in sport-specific field tests of female wheelchair basketball players (*n* = 13).

	Median	Minimum	Maximum	Range
Circumference				
Upper arm (relaxed) (cm)	27.2	20.5	38.6	18.1
Forearm (cm)	24.9	20.5	31.1	10.6
Wrist (cm)	15.5	13.9	18.5	4.6
Waist (cm)	79.0	64.5	114.0	49.5
Length/width/breath/depth				
Thigh length (cm)	38.0	29.0	39.5	10.5
Shoulder-elbow length (cm)	33.0	23.0	37.0	14.0
Elbow-wrist length (cm)	25.9	22.3	30.8	8.5
Elbow breadth (cm)	5.8	4.8	7.3	2.5
Wrist breadth (cm)	4.8	4.2	5.7	1.5
Transverse chest width (cm)	25.4	22.6	32.0	9.4
Anterior–posterior chest depth (cm)	17.3	15.6	22.8	7.2
SitH1 (cm)	127.0	115.0	147.0	32.0
SitH2 (cm)	168.0	145.0	185.0	40.0
Skinfolds and body composition				
Subscapular (mm)	13.4	10.5	32.0	21.5
Triceps (mm)	19.5	9.5	37.0	27.5
Biceps (mm)	8.2	3.6	21.0	17.4
Suprailiac (mm)	19.0	12.8	38.7	25.9
Body fat (%)	29.7	22.3	39.8	17.5
Performance in field tests				
5 m sprint (s)	2.3	1.7	3.2	1.5
20 m sprint with ball (s)	7.7	6.5	10.9	4.4
Suicide (s)	56.7	44.9	63.5	18.6
Lay-ups (n)	11	1	36	35
Pass for accuracy (n)	20	1	26	25
Maximal pass (m)	7.8	4	10.9	6.9
Spot-shot (n)	28	1	40	39

*SitH1, Sitting height; SitH2, maximal vertical reach from seated position.*

**Table 5:** Anthropometry and body composition of Point-matched female and male wheelchair basketball players.

Variable	Females (*n* = 13)	Males (*n* = 26)	*t*	*P*-value	Effect size	Power
Circumference						
Upper arm (relaxed) (cm)	28.0 ± 4.7	30.5 ± 4.0	-1.746	0.089	0.6	0.4
Forearm (cm)	24.6 ± 3.0	27.2 ± 2.8	-2.683	**0.011**	0.9	0.7
Wrist (cm)	15.6 ± 1.3	17.1 ± 1.0	-3.789	**0.001**	1.2	0.9
Waist (cm)	82.5 ± 14.4	93.5 ± 15.65	2.113	**0.041**	0.7	0.5
Length/width/breath/depth						
Thigh length (cm)	35.9 ± 3.6	38.9 ± 3.9	-2.335	**0.025**	0.8	0.6
Shoulder-elbow length (cm)	32.4 ± 3.4	35.7 ± 2.6	-3.404	**0.002**	1.1	0.9
Elbow-wrist length (cm)	25.8 ± 2.1	28.3 ± 1.8	-3.872	**<0.001**	1.3	1.0
Elbow breadth (cm)	5.9 ± 0.6	6.5 ± 0.5	-3.847	**<0.001**	1.2	0.9
Wrist breadth (cm)	4.8 ± 0.4	5.3 ± 0.5	-3.276	**0.002**	1.1	0.9
Transverse chest width (cm)	25.4 ± 2.5	28.7 ± 3.3	-3.146	**0.003**	1.1	0.9
Anterior–posterior chest depth (cm)	17.9 ± 2.4	20.2 ± 3.6	-2.147	**0.038**	0.8	0.6
SitH1 (cm)	129.0 ± 10.3	134.7 ± 10.5	-1.591	0.120	0.5	0.3
SitH2 (cm)	167.5 ± 11.9	177.0 ± 15.8	-1.913	0.064	0.7	0.4
Skinfolds and body composition						
Subscapular (mm)	17.4 ± 7.7	21.5 ± 11.2	-0.906	0.371	0.4	0.2
Triceps (mm)	18.6 ± 7.3	16.9 ± 7.8	0.638	0.527	0.2	0.1
Biceps (mm)	9.8 ± 5.6	8.1 ± 4.1	0.723	0.474	0.3	0.1
Suprailiac (mm)	22.6 ± 8.2	24.2 ± 8.6	-0.559	0.579	0.2	0.1
Body fat (%)	30.7 ± 6.0	23.2 ± 5.4	3.977	**<0.001**	1.3	1.0

*Student’s *t-*test. Data are means ± SD. Significant *P-*values are in bold. SitH1, sitting height; SitH2, maximal vertical reach from seated position; Power, *post hoc* power analysis.*

**FIGURE 2:**
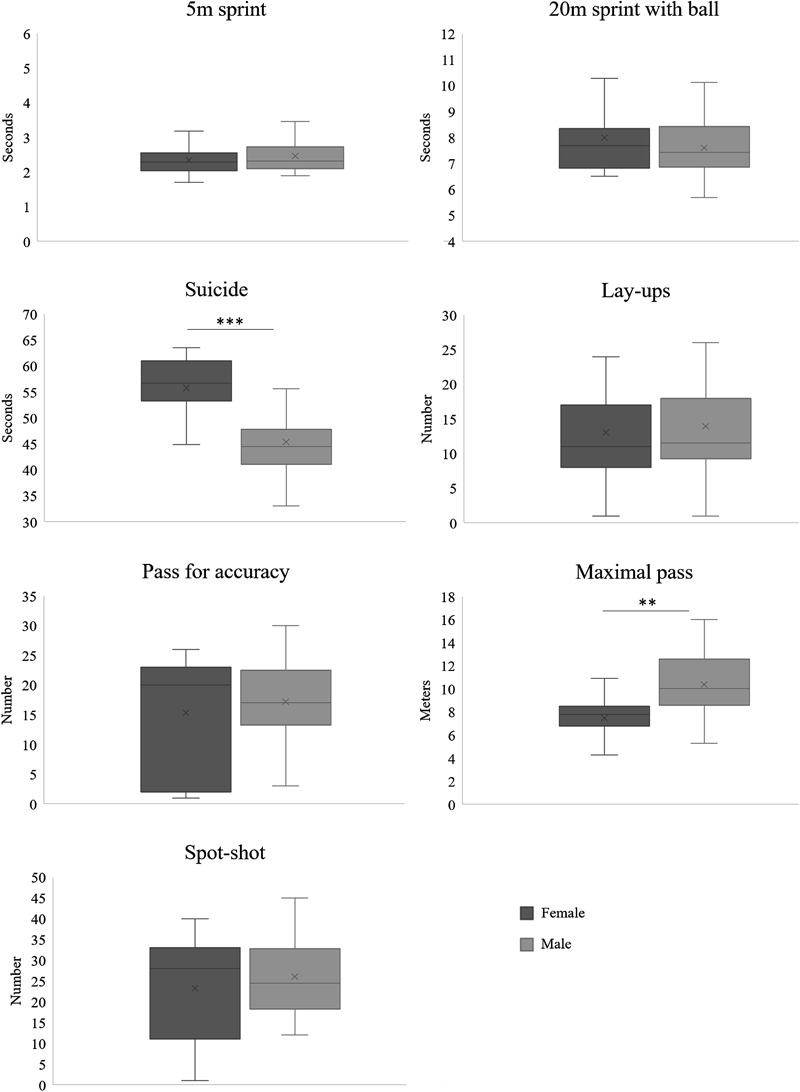
Performance in sport-specific field tests of classification-matched female (*n* = 13) and male (*n* = 26) wheelchair basketball (WB) players. ^∗∗^*P* = 0.002 [effect size (ES), 1.2; Power, 0.8.; ^∗∗∗^*P* < 0.001 (ES, 1.6; Power, 0.9) (paired-samples *t*-test, two-tailed)]. In the box plot, the upper horizontal line of the box represents the 75th percentile, the lower horizontal line of the box is the 25th percentile; the horizontal bar within the box is the median value; the x symbol within the box is the mean value; the upper and lower line outside the box represents minimum and maximum value, respectively (error bars).

The performance of the whole sample of female and male WB players in seven sport-specific field tests is reported in **Figure 2**. A significant difference between sexes was found in two tests namely, the maximal pass (*P* = 0.002, *d* = 1.2) and the suicide (*P* < 0.001, *d* = 1.6), test females performing worse than males in both tests. In the maximal pass test, males were able to pass the basketball ball on average 28% further than females. In the suicide test, males took an average 23% less time to complete the required distance.

In Subgroup A of WB players (see section “Materials and Methods”) females and males had similar age (24.8 ± 8.8 and 20.8 ± 3.5 years, respectively; *P* = 0.222, *d* = 0.6), self-reported duration of injury (12.6 ± 6.9 and 15.0 ± 7.0 years, respectively; *P* = 0.466, *d* = 0.3), and WB experience (5.0 ± 3.1 and 5.4 ± 2.5 years, respectively; *P* = 0.743, *d* = 0.1). In Subgroup A, females performed significantly worse (15.3%; *P* = 0.042, *d* = 1.1) than males in the suicide test(**Table 6**).

**Table 6:** Performance in sport-specific field tests of Subgroup A (see **Table 2** and see section “Materials and Methods”).

Sport-specific field test	Females (*n* = 9)	Males (*n* = 9)	*t*	*P*-value
5 m sprint (s)	2.1 ± 0.3	2.4 ± 0.6	-1.432	0.171
20 m sprint with ball (s)	7.8 ± 1.3	7.3 ± 0.9	0.979	0.342
Suicide (s)	54.2 ± 7.0	45.9 ± 8.7	2.214	**0.042**
Lay-ups (n)	15.8 ± 8.9	13.9 ± 5.1	0.552	0.589
Pass for accuracy (n)	16.7 ± 8.7	16.9 ± 7.5	0.376	0.548
Maximal pass (m)	8.1 ± 1.7	9.6 ± 2.8	-1.322	0.205
Spot-shot (n)	24.2 ± 13.0	21.8 ± 6.0	3.171	0.094

*Male players had 1.0 assigned Point less than females. Student’s *t-*test. Data are means ± SD. Significant *P*-values are in bold.*

In Subgroup B of WB players (see section “Materials and Methods”) females and males had similar age (27.2 ± 10.2 and 21.6 ± 7.1 years, respectively; *P* = 0.191, *d* = 0.6), self-reported duration of injury (12.6 ± 6.9, 16.4 ± 5.2 years, respectively; *P* = 0.195, *d* = 0.6) and WB experience (4.8 ± 2.9 and 6.0 ± 2.7 y, respectively; *P* = 0.368, *d* = 0.4). In Subgroup B, females performed similarly to males in all sport-specific field tests (**Table 7**).

**Table 7:** Performance in sport-specific field tests in Subgroup B (see **Table 3** and see section “Materials and Methods”).

Sport-specific field test	Females (*n* = 9)	Males (*n* = 9)	*t*	*P*-value
5 m sprint (s)	2.2 ± 0.4	2.6 ± 0.5	-2.097	0.052
20 m sprint with ball (s)	7.6 ± 1.2	8.1 ± 1.5	-0.756	0.460
Suicide (s)	53.9 ± 6.7	47.7 ± 8.0	1.786	0.093
Lay-ups (n)	16.3 ± 8.9	10.9 ± 6.1	1.513	0.150
Pass for accuracy (n)	16.2 ± 8.4	16.0 ± 8.5	0.560	0.956
Maximal pass (m)	8.2 ± 1.7	8.5 ± 1.7	-0.446	0.662
Spot-shot (n)	25.3 ± 13.7	24.1 ± 7.7	0.234	0.819

*Male players had 1.5 assigned Point less than females. Student’s *t*-test. Data are means ± SD.*

## Discussion

The assessment of physique and performance in athletes with disabilities is a very challenging area of study due to the limited total population of athletes and to the enormous variability within the population itself leading to large territorial spread for most studies. Further, males and females are currently grouped together in scientific studies thereby increasing variability and making direct comparison between studies difficult or even inaccurate.

This is the first study investigating anthropometry, body composition, and performance in sport-specific field tests of female WB players as well as exploring sex-related differences in the above variables. It might be deemed that the sample of WB players included in this study is small in absolute terms (*n* = 13); however, this figure represents about 40% of female WB players in Italy and should be considered representative enough of the reference population.

The results of this study showed that:

(1) The majority of female WB players are overweight.
(2) Female and male WB players differ in several upper body anthropometric variables and females show greater %FM.
(3) Sex-related differences exist in the performance of male and female WB players, which are mainly associated with passing (explosiveness) and resistance ability.
(4) Sex-related differences in performance might be fully compensated if 1.5 functional points are subtracted from female WB players.

### Anthropometry and Body Composition

A main finding of this study was that mean %FM in female WB players is 30.7% i.e., above the current cut-off for obesity. The body mass index (BMI) is commonly used to define obesity, despite it is not able to distinguish lean body mass from fat mass (Gallagher et al., 1996). Dual-energy X-ray absorptiometry (DXA) offers the opportunity for precise characterization of adiposity. According to several studies (Okorodudu et al., 2010; Shah and Braverman, 2012) using DXA, the cut-off level for obesity is percent body fat ≥30% for women and ≥25% for men. Therefore, our finding suggests that athletic disabled females tend to accumulate excess body fat. The current finding is in line with data in the wider population of subjects with physical disabilities where the reduced physical capacity in injured parts of the body deriving from immobilization and skeletal muscle denervation would result in muscle atrophy and an increase in subcutaneous and intramuscular fat mass (Talmadge et al., 2002; Maggioni et al., 2003; Gorgey and Dudley, 2007; Mojtahedi et al., 2009; Sutton et al., 2009; Collins et al., 2010; Gorgey and Shepherd, 2010; Beck et al., 2014). Data available in spinal cord injured subjects highlights that they suffer from excessive adiposity (%FM > 30%) (Spungen et al., 2003; Gorgey and Gater, 2011). When compared to gender, age, and BMI-matched able-bodied controls, individuals with spinal cord injury have greater total body fat mass (Kocina, 1997; Spungen et al., 2003; Clasey and Gater, 2005; McDonald et al., 2007; Gorgey et al., 2010), and more fat per unit of BMI (Spungen et al., 2003), especially in the lower limbs (Spungen et al., 2000, 2003; Maggioni et al., 2003; Modlesky et al., 2004). Nevertheless, practicing WB may help disabled females in limiting body fat accrual. In fact, it has been shown that in sedentary females with traumatic, complete motor paraplegia for at least 2 years DXA-measured %FM was 45.5 ± 10.2% (Beck et al., 2014). The mean value of waist circumference in female WB players was 82.5 ± 14.4 cm (**Table 5**) that is greater than the cut-off point for increased health risk in Europid females (i.e., ≥80 cm; World Health Organization [WHO], 2000). Moreover, in 38% of female WB players the waist circumference was equal or above 80 cm suggesting an increased risk of metabolic complications associated with obesity (World Health Organization [WHO], 2000; Alberti et al., 2007). Further, the ratio of the subscapular to the triceps skinfolds in WB female athletes was 0.96 ± 0.29 and the ratio of waist circumference to arm circumference was 2.96 ± 0.22 also indicating a central pattern of adiposity. This altered distribution of adipose tissue was expected and is in accordance with previous findings (Wilmet et al., 1995; Spungen et al., 2000; Beck et al., 2014). Our findings could be explained, at least in part, by the fact that most female WB players were wheelchair-dependent and, therefore, they had to use their arms to transfer themselves and to propel the wheelchair; these activities possibly contributed to lower adiposity in upper extremity thereby affecting the peripheral-to-central fat distribution. When compared with male WB players, females showed a significantly (*P* < 0.001) higher %BF (∼8%). This is consistent with able-bodied literature in this field (Kirchengast, 2002; Wells, 2007) as well as findings in spinal cord injured sedentary males (Beck et al., 2014) who had a ∼10% lower DXA-measured fat mass versus their female counterparts suggesting that the well-known larger adiposity of females vs. males is maintained in the wheelchair population. When interpreting our data, it is important to bear in mind that we estimated %FM with an anthropometric equation developed in able-bodied subjects (Durnin and Womersley, 1974). Preliminary results from our laboratory showed that this equation underestimated %FM in spinal cord-injured athletes by about 3% using DXA as the reference. Future studies will therefore benefit from the use of more accurate methods to assess %FM in the disabled athletic population.

In our sample of female and male WB players, both average SitH1 and SitH2 were higher in males (+4.2 and +5.4%, respectively), but the difference was not significant (SitH1, *P* = 0.12) or borderline significant (SitH2, *P* = 0.06). Stature has long been appreciated as a key body parameter in running basketball performance (Cormery et al., 2008) and, in able-bodied basketball, adult males are on average 7% taller than females (Kirchengast, 2002). While in the able-bodied population the standing height is proportional to sitting height (Cotes et al., 2009), in subjects with disabilities the sitting height is in part associated with stature and in part with impairment. In fact, the type and degree of physical impairment makes erect sitting problematic and WB players showing more severe impairments adopt a deeper-seated position on the basketball wheelchair to gain stability and balance. This limits the trunk motion and, consequently reduces SitH2. In addition to a lower sitting position in the basketball wheelchair, male and female players assigned a lower Point would possess less trunk and abdominal strength compared to players assigned a higher Point. In the absence of statistically significant differences in SitH1 and SitH2 between male and female players, inference on the effect of these variables on performance is limited. However, non-impairment-affected height-related variables namely, the shoulder-elbow, elbow-wrist, and thigh length were significantly greater in males vs. females by 9.3, 8.8, and 7.8%, respectively, (**Table 5**) suggesting that males were actually taller than females. Apart from the sitting height, a longer upper arm in males could facilitate passing and wheelchair maneuvering by giving the players, whose body is constrained in the wheelchair, more room to move the ball while making a pass or while propelling the wheelchair.

### Performance in Sport-Specific Field Tests

Wheelchair basketball is an intermittent sport characterized by a combination of repeated high intensity sprints and rapid acceleration and deceleration (Wang et al., 2005; Goosey-Tolfrey, 2006). To display good performance during the game, WB players need to master and perform several skills simultaneously for wheelchair maneuvering (i.e., propulsion, starting and stopping and changing wheelchair direction) and ball handling (i.e., shooting, passing, dribbling, or rebounding). Obviously, performance is also affected by residual functional potential. In elite WB athletes, some limited sex-related differences in team scores were found (Vanlandewijck et al., 2004; Molik et al., 2009), male WB players being more accurate than females in field-goal and free-throw shooting (Vanlandewijck et al., 2004). In the current study, we expand on previous data by investigating the performance of female and male WB players in a battery of seven sport-specific field tests (De Groot et al., 2012; Cavedon et al., 2015) assessing different skills required in the WB game. To minimize the possible effect of residual functional potential on the scores, female and male players were matched according to the players’ assigned Point. Also, the female and male groups resulted similar in terms of age, duration of injury, WB experience, and weekly amount of training thereby making comparison more reliable.

Results showed that, on average, males performed better than females in all sport-specific field tests but for the 5 m sprint test; however, males performed significantly better than females in the maximal pass and the suicide tests. These two tests mainly explore explosiveness in passing and resistance, respectively. In the maximal pass test, males shot the basketball ball ∼28% further than females; in the suicide test males took ∼23% less time to complete the required distance vs. females. This could be related to lesser muscle strength and endurance along with lower levels of cardiovascular fitness in females irrespective of the degree and the severity of the impairment. Such a suggestion is supported by the presence of an innate sex disparity in the relative strength and muscular endurance features of the muscles of the upper body in able-bodied subjects (Miller et al., 1993). Similarly, Padulo et al. (2016) showed that males are faster than females in the repeated sprint ability tests.

An optimal body mass has been shown to be related to success in running basketball (Apostolidis et al., 2004; Nikolaidis et al., 2015). Therefore, it could be argued that better performance in the suicide test in male vs. female WB players is explained at least in part by the higher %FM found in the latter. Indeed, Apostolidis et al. (2004) reported a negative correlation between %FM and performance time in running speed and high intensity shuttle run; more recently, Nikolaidis et al. (2015) found that an excess body mass has a negative effect on sprinting ability in young basketball players. It is however important to bear in mind that, despite similarities exist between WB and running basketball, in disable WB athletes excess %FM could not have the same implications on sport performance as in their able-bodied counterpart. In fact, in able-bodied basketball players fat mass is an extra load acting as a dead weight to be moved whereas in WB players have to carry their body weight in a wheelchair during play and do not have to jump. Accordingly, WB players may not be hindered by extra body weight as much as able-bodied players. Future research is needed to clarify the relationship between %FM and WB performance in WB players with different %FM profiles (e.g., normal weight or overweight players).

In a previous study investigating younger male and female WB players (Cavedon et al., 2015), better performance in sport-specific field tests was associated with higher scores in game-related statistics. In particular, the maximal pass test was shown to be a strong predictor of the number of field goals and the number of total points scored by a player per match. The same study also reported a weaker, albeit significant association between the suicide test (exploring speed and endurance) and game-related statistics. Based on the above, it could be hypothesized that being male positively affects game-related statistics, thereby favoring mixed teams, which include a larger number of male WB players. Further work is therefore needed to quantify sex-related differences in game-related statistics when males and females compete together.

In Subgroup A (**Table 6**), female (median point score 2.5 points) and male (median Point score 1.5 points) WB players showed similar performance in all sport-specific field tests, but for the suicide test. This suggests that sex-related differences in sport-specific field tests where not fully compensated by subtracting 1.0 point from each female WB player. In Subgroup B (**Table 7**), female (median point score 2.5 points) and male (median point score of 1.0) WB players showed similar performance in all sport-specific field tests. This indicates that sex-related differences in sport-specific field tests are compensated in full by subtracting 1.5 points from each female WB players. These findings may impact on current provisions adopted to promote fair and equitable competitions in championships where mixed (M/F) teams are allowed to compete.

### Study Limitations and Strengths

This study has limitations that should be mentioned. First, we were not able to match each female player with a male from the same team. Therefore, we could not account for possible differences in training programs of male and female players belonging to different teams. Second, despite similar WB experience and volume of training in the female and male groups, matched players could have been playing at different competitive levels with possible differences in the development of their WB skills. Third, the overall in-season on court playing time was not available for all players, thereby preventing assessment of the possible effect of this variable on performance in sport-specific field tests.

In this work, however, there are also a number of strengths to be highlighted. First, the data provided in this study help to fill some important gaps in the literature providing better understanding of the relationship between physique and performance in female WB players with important practical implications from the perspective of both training and the classification system. Second, our finding may have an important impact on the classification system in countries where males and females play together in mixed teams.

## Conclusion

This study provided new data on the physique and performance of female WB players. The presence of sex-related differences in field test performance of WB players should stimulate coaches and trainers to develop and optimize training programs to take into account sex-related differences in WB skill proficiency as well as to personalize training according to sex and to identify training priorities in mixed teams. In particular, where male and female players train and compete together, sex-specific strength and endurance training should be carefully considered. The present study also addressed the validity of the current WB classification system thereby promoting an “evidence-based classification system through research” (International Paralympic Committee [IPC], 2007, item 15.2.2) and opens new understanding into the current classification system of WB players. The results demonstrated a clear difference in performance between male and female players, suggesting that when male and female athletes compete together on the same team a correct system of classification is important to assure fair and equitable competition. A valid classification system should therefore take into account both the impairment and the impact of the player’s sex on performance. Taken together, our results suggest that a 1.5-point subtraction for female players is fairer in order to match the real difference in performance between male and female WB players in mixed teams. The promotion of an appropriate evidence-based classification system for athletes based on both sex and functional ability would encourage and facilitate the participation in WB of male and female players in mixed teams in a fair and unbiased structure.

## Author Contributions

VC, CZ, and CM conceived and designed the experiments, performed the experiments, analyzed the data, and wrote the paper.

## Conflict of Interest Statement

The authors declare that the research was conducted in the absence of any commercial or financial relationships that could be construed as a potential conflict of interest.

## References

[B1] Alberti K. G. M. M. Zimmet P. Shaw J. (2007). International diabetes federation: a consensus on type 2 diabetes prevention. *Diabet. Med.* 24 451–463. 10.1111/j.1464-5491.2007.02157.x 17470191

[B2] Apostolidis N. Nassis G. P. Bolatoglou T. Geladas N. D. (2004). Physiological and technical characteristics of elite young basketball players. *J. Sports Med. Phys. Fitness* 44 157–163. 15470313

[B3] Beck L. A. Lamb J. L. Atkinson E. J. Wuermser L. A. Amin S. (2014). Body composition of women and men with complete motor paraplegia. *J. Spinal Cord Med.* 37 359–365. 10.1179/2045772313Y.0000000151 24090208 PMC4116716

[B4] Bernardi M. Carucci S. Faiola F. Egidi F. Marini C. Castellano V. (2012). Physical fitness evaluation of paralympic winter sports sitting athletes. *Clin. J. Sport Med.* 22 26–30. 10.1097/JSM.0b013e31824237b5 22222593

[B5] Bernardi M. Guerra E. Di Giacinto B. Di Cesare A. Castellano V. Bhambhani Y. (2010). Field evaluation of paralympic athletes in selected sports: implications for training. *Med. Sci. Sports Exerc.* 42 1200–1208. 10.1249/MSS.0b013e3181c67d82 19997027

[B6] Box G. E. P. Cox D. R. (1964). An analysis of transformations. *J. R. Stat. Soc. Series B Stat. Methodol.* 26 211–252.

[B7] Cavedon V. Zancanaro C. Milanese C. (2015). Physique and performance of young wheelchair basketball players in relation with classification. *PLoS One* 10:e0143621. 10.1371/journal.pone.0143621 26606681 PMC4659662

[B8] Clasey J. L. Gater D. R. Jr. (2005). A comparison of hydrostatic weighing and air displacement plethysmography in adults with spinal cord injury. *Arch. Phys. Med. Rehabil.* 86 2106–2113. 10.1016/j.apmr.2005.06.013 16271556

[B9] Cohen J. (1988). *Statistical Power Analysis for the Behavioral Sciences* 2nd Edn. Hillsdale, NJ: Lawrence Erlbaum Associates.

[B10] Collins E. G. Gater D. Kiratli J. Butler J. Hanson K. Langbein W. E. (2010). Energy cost of physical activities in persons with spinal cord injury. *Med. Sci. Sports Exerc.* 42 691–700. 10.1249/MSS.0b013e3181bb902f 19952846

[B11] Connick M. J. Beckman E. Ibusuki T. Malone L. Tweedy S. M. (2016). Evaluation of methods for calculating maximum allowable standing height in amputees competing in Paralympic athletics. *Scand. J. Med. Sci. Sports.* 26 1353–1359. 10.1111/sms.12586 26589580

[B12] Cormery B. Marcil M. Bouvard M. (2008). Rule change incidence on physiological characteristics of elite basketball players: a 10-year-period investigation. *Br. J. Sports Med.* 42 25–30. 10.1136/bjsm.2006.033316 17526624

[B13] Cotes J. E. Chinn D. J. Miller M. R. (2009). *Lung Function: Physiology, Measurement and Application in Medicine*. Hoboken, NJ: Blackwell publishing.

[B14] Curtis K. A. Black K. (1999). Shoulder pain in female wheelchair basketball players. *J. Orthop. Sports Phys. Ther.* 29 225–231. 10.2519/jospt.1999.29.4.225 10322595

[B15] De Groot S. Balvers I. J. M. Kouwenhoven S. M. Janssen T. W. J. (2012). Validity and reliability of tests determining performance-related components of wheelchair basketball. *J. Sports Sci.* 30 879–887. 10.1080/02640414.2012.675082 22489567

[B16] De Lira C. A. B. Vancini R. L. Minozzo F. C. Sousa B. S. Dubas J. P. Andrade M. S. (2010). Relationship between aerobic and anaerobic parameters and functional classification in wheelchair basketball players: wheelchair basketball. *Scand. J. Med. Sci. Sports* 20 638–643. 10.1111/j.1600-0838.2009.00934.x 19793219

[B17] De Witte A. M. H. Hoozemans M. J. M. Berger M. A. M. van der Woude L. H. V. Veeger D. (2015). Do field position and playing standard influence athlete performance in wheelchair basketball? *J. Sports Sci.* 34 811–820. 10.1080/02640414.2015.1072641 26222201

[B18] Dingley A. A. Pyne D. B. Burkett B. (2015). Relationships between propulsion and anthropometry in paralympic swimmers. *Int. J. Sports Physiol. Perform.* 10 978–985. 10.1123/ijspp.2014-0186 25756388

[B19] Durnin J. V. Womersley J. (1974). Body fat assessed from total body density and its estimation from skinfold thickness: measurements on 481 men and women aged from 16 to 72 Years. *Br. J. Nutr.* 32 77–97. 10.1079/BJN19740060 4843734

[B20] Faul F. Erdfelder E. Buchner A. Lang A. G. (2009). Statistical power analyses using G^∗^Power 3.1: tests for correlation and regression analyses. *Behav. Res. Methods* 41 1149–1160. 10.3758/BRM.41.4.1149 19897823

[B21] Gallagher D. Visser M. Sepulveda D. Pierson R. N. Harris T. Heimsfield S. B. (1996). How useful is body mass index for comparison of body fatness across age, sex, and ethnic groups? *Am. J. Epidemiol.* 143 228–239. 10.1093/oxfordjournals.aje.a008733 8561156

[B22] Gómez M. A. Molik B. Morgulec-Adamowicz N. Szyman R. (2015). Performance analysis of elite women’s wheelchair basketball players according to team-strength, playing-time and players’ classification. *Int. J. Perform. Anal. Sport* 15 268–283. 10.1080/24748668.2015.11868792

[B23] Gómez M. A. Pérez J. Molik B. Szyman R. J. Sampaio J. (2014). Performance analysis of elite men’s and women’s wheelchair basketball teams. *J. Sports Sci.* 32 1066–1075. 10.1080/02640414.2013.879334 24506819

[B24] Goosey-Tolfrey V. (2006). Aerobic capacity and peak power output of elite quadriplegic games players. *Br. J. Sports Med.* 40 684–687. 10.1136/bjsm.2006.026815 16611721 PMC2579453

[B25] Gore C. Norton K. Olds T. (2000). “Acreditación en antropometría: un modelo australiano,” in *Antropométrica* eds Norton K. Olds T. (Rosario: Biosystem Servicio Educativo) 263–272.

[B26] Gorgey A. S. Chiodo A. E. Zemper E. D. Hornyak J. E. Rodriguez G. M. Gater D. R. (2010). Relationship of spasticity to soft tissue body composition and the metabolic profile in persons with chronic motor complete spinal cord injury. *J. Spinal Cord Med.* 33 6–15. 10.1080/10790268.2010.11689669 20397439 PMC2853330

[B27] Gorgey A. S. Dudley G. A. (2007). Skeletal muscle atrophy and increased intramuscular fat after incomplete spinal cord injury. *Spinal Cord* 45 304–309. 10.1038/sj.sc.3101968 16940987

[B28] Gorgey A. S. Gater D. R. (2011). A preliminary report on the effects of the level of spinal cord injury on the association between central adiposity and metabolic profile. *PM R* 3 440–446. 10.1016/j.pmrj.2011.01.011 21570032

[B29] Gorgey A. S. Shepherd C. (2010). Skeletal muscle hypertrophy and decreased intramuscular fat after unilateral resistance training in spinal cord injury: case report. *J. Spinal Cord Med.* 33 90–95. 10.1080/10790268.2010.11689681 20397451 PMC2853337

[B30] Granados C. Yanci J. Badiola A. Iturricastillo A. Otero M. Olasagasti J. (2015). Anthropometry and performance in wheelchair basketball. *J. Strength Cond. Res.* 29 1812–1820. 10.1519/JSC.0000000000000817 25536537

[B31] International Paralympic Committee [IPC] (2007). *IPC Classification Code and International Standards*. Bonn: International Paralympic Committee.

[B32] International Wheelchair Basketball Federation [IWBF] (2014). *Official Wheelchair Basketball Rules*. Available at: https://iwbf.org/wp-content/uploads/2016/08/2014_IWBF_Rules_V2.pdf

[B33] Italian Wheelchair Basketball Federation (2017). *Young Wheelchair Basketball Championship-Roberto Marson’s Trophy. Rules, Activity Organization, Functional Classifications*. Available at: https://www.federipic.it/normative/regolamento-campionato-giovanile

[B34] Italian Wheelchair Basketball Federation (2014). *Vademecum-Competitive Season 2013/2014*.

[B35] Kirchengast S. (2002). Sex differences in body composition are detectable well before puberty. *Humanbiol. Budapest.* 27 121–128.

[B36] Kocina P. (1997). Body composition of spinal cord injured adults. *Sports Med.* 23 48–60. 10.2165/00007256-199723010-00005 9017859

[B37] Lohman T. G. Roche A. F. Martorell R. (1988). *Anthropometric Standardization Reference Manual*. Champaign, IL: Human Kinetics Press.

[B38] Maggioni M. Bertoli S. Margonato V. Merati G. Veicsteinas A. Testolin G. (2003). Body composition assessment in spinal cord injury subjects. *Acta Diabetol.* 40 183–186. 10.1007/s00592-003-0061-7 14618468

[B39] Malone L. A. Gervais P. L. Steadward R. D. (2002). Shooting mechanics related to player classification and free throw success in wheelchair basketball. *J. Rehabil. Res. Dev.* 39 701–719. 17943672

[B40] McDonald C. M. Abresch-Meyer A. L. Nelson M. D. Widman L. M. (2007). Body mass index and body composition measures by dual x-ray absorptiometry in patients aged 10 to 21 years with spinal cord injury. *J. Spinal Cord Med.* 30 97–104. 10.1080/10790268.2007.11754612 17874694 PMC2031982

[B41] Miller A. E. MacDougall J. D. Tarnopolsky M. A. Sale D. G. (1993). Gender differences in strength and muscle fiber characteristics. *Eur. J. Appl. Physiol.* 66 254–262. 10.1007/BF00235103 8477683

[B42] Modlesky C. M. Bickel C. S. Slade J. M. Meyer R. A. Cureton K. J. Dudley G. A. (2004). Assessment of skeletal muscle mass in men with spinal cord injury using dual-energy X-ray absorptiometry and magnetic resonance imaging. *J. Appl. Physiol.* 96 561–565. 10.1152/japplphysiol.00207.2003 14527962

[B43] Mojtahedi M. C. Valentine R. J. Evans E. M. (2009). Body composition assessment in athletes with spinal cord injury: comparison of field methods with dual-energy X-ray absorptiometry. *Spinal Cord* 47 698–704. 10.1038/sc.2009.20 19290014

[B44] Molik B. Kosmol A. Morgulec-Adamowicz N. Laskin J. J. Jezior T. Patrzałek M. (2009). Game efficiency of elite female wheelchair basketball players during world championships (Gold Cup) 2006. *Eur. J. Adapt. Phys. Act* 2 26–38.

[B45] Molik B. Laskin J. J. Kosmol A. Skucas K. Bida U. (2010). Relationship between functional classification levels and anaerobic performance of wheelchair basketball athletes. *Res. Q. Exerc. Sport* 81 69–73. 10.1080/02701367.2010.10599629 20387400

[B46] Molik B. Morgulec-Adamowicz N. Kosmol A. Yilla A. B. Filipkowska A. Lewandowski M. (2012). Game performance in ice sledge hockey: an exploratory examination into type of disability and anthropometric parameters. *Clin. J. Sport Med.* 22 65–69. 10.1097/JSM.0b013e3182420677 22222590

[B47] Nikolaidis P. T. Asadi A. Santos E. J. Calleja-González J. Padulo J. Chtourou H. (2015). Relationship of body mass status with running and jumping performances in young basketball players. *Muscles Ligaments Tendons J.* 5 187–194. 10.11138/mltj/2015.5.3.187 26605193 PMC4617219

[B48] Okorodudu D. O. Jumean M. F. Montori V. M. Romero-Corral A. Somers V. K. Erwin P. J. (2010). Diagnostic performance of body mass index to identify obesity as defined by body adiposity: a systematic review and meta-analysis. *Int. J. Obes.* 34 791–799. 10.1038/ijo.2010.5 20125098

[B49] Padulo J. Bragazzi N. L. Nikolaidis P. T. Dello Iacono A. Attene G. Pizzolato F. (2016). Repeated sprint ability in young basketball players: multi-direction vs. one-change of direction (part 1). *Front. Physiol.* 7:133. 10.3389/fphys.2016.00133 27148072 PMC4840326

[B50] Porto Y. C. Almeida M. de Sá C. K. Schwingel P. A. Zoppi C. C. (2008). Anthropometric and physical characteristics of motor disabilited paralympic rowers. *Res. Sports Med.* 16 203–212. 10.1080/15438620802103437 18785062

[B51] Shah N. R. Braverman E. R. (2012). Measuring adiposity in patients: the utility of Body Mass Index (BMI), percent body fat, and Leptin. *PLoS One* 7:e33308. 10.1371/journal.pone.0033308 22485140 PMC3317663

[B52] Siri W. E. (1961). “Body composition from fluid space and density,” in *Techniques for Measuring Body Composition* eds Brozek J. Henschel A. (Washington, DC: National Academy of Science).

[B53] Spathis J. G. Connick M. J. Beckman E. M. Newcombe P. A. Tweedy S. M. (2015). Reliability and validity of a talent identification test battery for seated and standing Paralympic throws. *J. Sports Sci.* 33 863–871. 10.1080/02640414.2014.969294 25371965

[B54] Spungen A. M. Adkins R. H. Stewart C. A. Wang J. Pierson R. N. Jr. Waters R. L. (2003). Factors influencing body composition in persons with spinal cord injury: a cross-sectional study. *J. Appl. Physiol.* 95 2398–2407. 10.1152/japplphysiol.00729.2002 12909613

[B55] Spungen A. M. Wang J. Pierson R. N. Jr. Bauman W. A. (2000). Soft tissue body composition differences in monozygotic twins discordant for spinal cord injury. *J. Appl. Physiol.* 88 1310–1315. 10.1152/jappl.2000.88.4.1310 10749824

[B56] Sutton L. Wallace J. Goosey-Tolfrey V. Scott M. Reilly T. (2009). Body composition of female wheelchair athletes. *Int. J. Sports Med.* 30 259–265. 10.1055/s-0028-1105941 19288390

[B57] Talmadge R. J. Roy R. R. Caiozzo V. J. Edgerton V. R. (2002). Mechanical properties of rat soleus after long-term spinal cord transection. *J. Appl. Physiol.* 93 1487–1497. 10.1152/japplphysiol.00053.2002 12235051

[B58] Ulijaszek S. J. Lourie J. A. (1994). “Intra- and inter-observer error in anthropometric measurement,” in *Anthropometry: The Individual and the Population* eds Ulijaszek S. J. Mascie-Taylor C. G. N. (Cambridge: Cambridge University Press). 10.1017/CBO9780511600500

[B59] Vanlandewijck Y. C. Evaggelinou C. Daly D. J. Verellen J. Van Houtte S. Aspeslagh V. (2004). The relationship between functional potential and field performance in elite female wheelchair basketball players. *J. Sports Sci.* 22 668–675. 10.1080/02640410310001655750 15370498

[B60] Vanlandewijck Y. C. Spaepen A. J. Lysens R. J. (1994). Wheelchair propulsion: functional ability dependent factors in wheelchair basketball players. *Scand. J. Rehabil. Med.* 26 37–48. 8023084

[B61] Wang Y. T. Chen S. Limroongreungrat W. Change L. S. (2005). Contributions of selected fundamentals factors to wheelchair basketball performance. *Med. Sci. Sports Exerc.* 3 130–137. 10.1249/01.MSS.0000150076.36706.B2 15632679

[B62] Weissland T. Faupin A. Borel B. Leprêtre P. M. (2015). Comparison between 30-15 intermittent fitness test and multistage field test on physiological responses in wheelchair basketball players. *Front. Physiol.* 6:380. 10.3389/fphys.2015.00380 26733875 PMC4679906

[B63] Wells J. C. K. (2007). Sexual dimorphism of body composition. *Best Pract. Res. Clin. Endocrinol. Metab.* 21 415–430. 17875489 10.1016/j.beem.2007.04.007

[B64] Wilmet E. Ismail A. A. Heilporn A. Welraeds D. Bergmann P. (1995). Longitudinal study of the bone mineral content and of soft tissue composition after spinal cord section. *Paraplegia* 33 674–677. 10.1038/sc.1995.141 8584304

[B65] World Health Organization [WHO] (2000). *Obesity: Preventing and Managing the Global Epidemic.* Report of a WHO Consultation (Technical Report Series 894. p. 11). Available at: http://whqlibdoc.who.int/trs/who_trs_894.pdf 11234459

